# Plasma concentrations of buprenorphine administered via matrix-type transdermal patches applied at three different anatomical locations in healthy adult horses

**DOI:** 10.3389/fpain.2024.1390322

**Published:** 2024-06-19

**Authors:** Vaidehi V. Paranjape, Heather K. Knych, Londa J. Berghaus, Shyla Giancola, Jessica Cathcart, Rachel A. Reed

**Affiliations:** ^1^ Department of Small Animal Clinical Sciences, Virginia-Maryland College of Veterinary Medicine, Virginia Polytechnic Institute and State University, Blacksburg, VA, United States; ^2^ K. L. Maddy Equine Analytical Pharmacology Laboratory, School of Veterinary Medicine, University of California-Davis, Davis, CA, United States; ^3^ Department of Large Animal Medicine, College of Veterinary Medicine, University of Georgia, Athens, GA, United States

**Keywords:** opioids, equine, analgesia, pain, pharmacology, gaskin, tail base, metacarpus

## Abstract

**Background:**

Anatomical location-dependent differences in transdermal opioid penetration are well described in human patients. Although this has been investigated in horses with fentanyl, there is no literature available on location-dependent plasma buprenorphine concentrations when administered as a transdermal matrix-type patch.

**Objective:**

This study aims to compare the plasma concentrations achieved from the matrix-type transdermal buprenorphine patches placed at different anatomical sites (metacarpus, gaskin, and ventral tail base) in healthy adult horses.

**Study design:**

This is a randomized experimental study with a Latin square design.

**Methods:**

Six adult horses were given each of three treatments with a minimum 10-day washout period. For each treatment, two 20 μg h^−1^ matrix-type buprenorphine patches were applied to the ventral aspect of the tail base (Tail_TDP_), metacarpus region (Metacarpus_TDP_), or gaskin region (Gaskin_TDP_). Whole blood samples (for determination of buprenorphine concentration) and physiological variables were collected before (0 h) and at 0.5, 2, 4, 6, 8, 10, 12, 16, 24, 32, 48, 56, 72, 96 and 120 h after patches were applied. The patches were removed 96 h following placement and were analyzed for residual buprenorphine content. Buprenorphine concentrations were measured in plasma by LC-MS/MS. A mixed-effects model was used to analyze the physiological variables.

**Results:**

Between the three treatment groups, there was no change in physiological variables across timepoints as compared to baseline and when compared to each other in a single horse and between horses (*p* > 0.3). When comparing all three locations, the buprenorphine uptake was observed to be more consistent with respect to measurable plasma concentrations >0.1 ng ml^−1^ when applied to the ventral aspect of the tail base. In the Tail_TDP_ group, the mean plasma buprenorphine concentrations were >0.1 ng ml^−1^ from 2 to 32 h. The highest group mean was 0.25 ng ml^−1^ noted at 4 h.

**Conclusions:**

The metacarpal and gaskin regions presented more erratic and inconsistent buprenorphine uptake and plasma concentrations as compared to the ventral aspect of the tail base. Further research must be directed at investigating the optimal dose, achievable duration of analgesia, change in measurable plasma concentrations, and behavioral and systemic effects.

## 1 Introduction

In the past decade, effective pain management in horses has become feasible thanks to research involving various analgesic drugs along with the development of pain scales allowing recognition of overt pain behaviors, changes in facial expressions and head position, and patients’ response to palpation and human interaction. The clinical impact of these studies is to enhance the well-being and welfare of this species by optimizing treatment strategies for pain based on severity and chronicity and utilizing multimodal analgesic regimes. Equine clinicians use various pharmaceutical classes to treat pain but the drug selection and route of administration is limited by some considerations specific to horses. Opioids are the most effective analgesics and are the mainstay of perioperative analgesia for treating pain in human and veterinary medicine. Injectable pure µ-receptor opioid agonists such as morphine, hydromorphone, and methadone are routine choices to treat perioperative pain in horses. However, clinicians hesitate to use this drug class in horses due to the apparent narrow margin between analgesia and excitation or arousal, gastrointestinal hypomotility, and challenges posed in quantifying consistent analgesic effects (1, 2).

The transdermal therapeutic system has also been assessed in horses for synthetic µ-opioid agonists such as fentanyl due to the advantage of (i) providing non-invasive, continuous pain control for extended periods; (ii) preventing wide variations in serum drug concentrations; (iii) reducing severity of adverse effects associated with repeated post-dose peaks in plasma concentration as seen with an injectable route; (iv) avoiding end-of-dose breakthrough pain; and (v) preventing first-pass metabolism occurring commonly with an oral route of administration (3, 4). Buprenorphine is another opioid that is available for transdermal drug delivery via patch application.

Buprenorphine is a semi-synthetic, highly lipophilic oripavine derivative that is classified as a high-affinity partial µ-receptor agonist and a κ-receptor antagonist that displays slow-dissociation kinetics. Its affinity for the opiate receptor is double, and its potency is approximately 30 times higher than morphine. Its therapeutic response lasts much longer than other opioids, and it has a wider safety profile. The partial agonism at the µ-receptor is a unique feature of buprenorphine and is attributed to its many distinctive properties, specifically that its analgesic effects plateau at higher doses, and ceiling effects on respiratory depression occur, which makes it safer than pure agonists of the µ-receptor (5–7). A transdermal matrix patch buprenorphine formulation, which was initially developed for human use, has been investigated for extra-label purposes in dogs (8–11), cats (12), pigs (13, 14), sheep (15, 16), and primates (17). Several equine studies report the clinical utility of injectable buprenorphine (i.e., intravenous, intramuscular, subcutaneous, and sublingual) to treat mild to moderate pain (18–24), increase nociceptive threshold (21–23), and offer superior-long lasting antinociception in comparison to butorphanol (24). However, there is minimal literature available on the use of buprenorphine via transdermal patch in horses (25, 26).

In horses, the ventral aspect of the tail is a common location for a transdermal patch system since the location is easily accessible, the application is easy, the patches can be secured, and contact with the skin can be maintained by covering the patch with an adhesive tape (27). It is crucial to understand that not only is the ease of application an important factor but so are the onset and duration of action and achievable plasma concentrations. The prediction of plasma concentrations is difficult with a transdermal route of administration due to the variability in drug absorption and systemic availability across species that can be influenced by the location of the patch (27–32). The objective of the present study was to compare the plasma concentrations achieved from the matrix-type transdermal buprenorphine patches placed at different anatomical locations (metacarpus, gaskin, and ventral tail base) in healthy adult horses. We hypothesized that the absorption of buprenorphine from the ventral tail base would be most reliable and yield consistent, quantifiable, and clinically relevant plasma concentrations.

## 2 Material and methods

### 2.1 Ethics statement

This study was approved by the University of Georgia Institutional Animal Care and Use Committee (animal use protocol: A2021 06-011).

### 2.2 Study animals

Six, university-owned adult, healthy horses (four mares and two geldings) aged 19 ± 7 years and weighing 559 ± 58 kg were enrolled in this prospective, Latin square study design. The animals were deemed healthy based on clinical history, thorough physical examination, and a normal complete blood count and biochemistry profile. The horses were housed in 3.65 m × 4.26 m stalls for acclimatization 16–20 h prior to treatment administration on each occasion. During the entire duration of the study when the horses were housed in this research environment, they were provided with 0.7 kg of senior feed (senior formula; Seminole Feed, Ocala, FL, USA) and 2–3 flakes of timothy hay twice daily with *ad libitum* access to water. On the same day, i.e., the day of arrival at the facility, a 14-gauge, 13 cm intravenous catheter (DayCath; MILA International, Florence, KY, USA) was placed aseptically in the cranial region of the jugular vein on the selected side for blood collection for pharmacokinetic analysis. The horses were then weighed, and a physical examination was performed to record the baseline heart rate (HR), respiratory rate (RR), and rectal temperature (Temp_rectal_). The catheter was periodically flushed with saline (0.9% sodium chloride; Baxter International Inc., Deerfield, IL, USA) and was monitored closely for blood clots and patency.

### 2.3 Treatment groups and transdermal buprenorphine patch application

**Figure 1:**
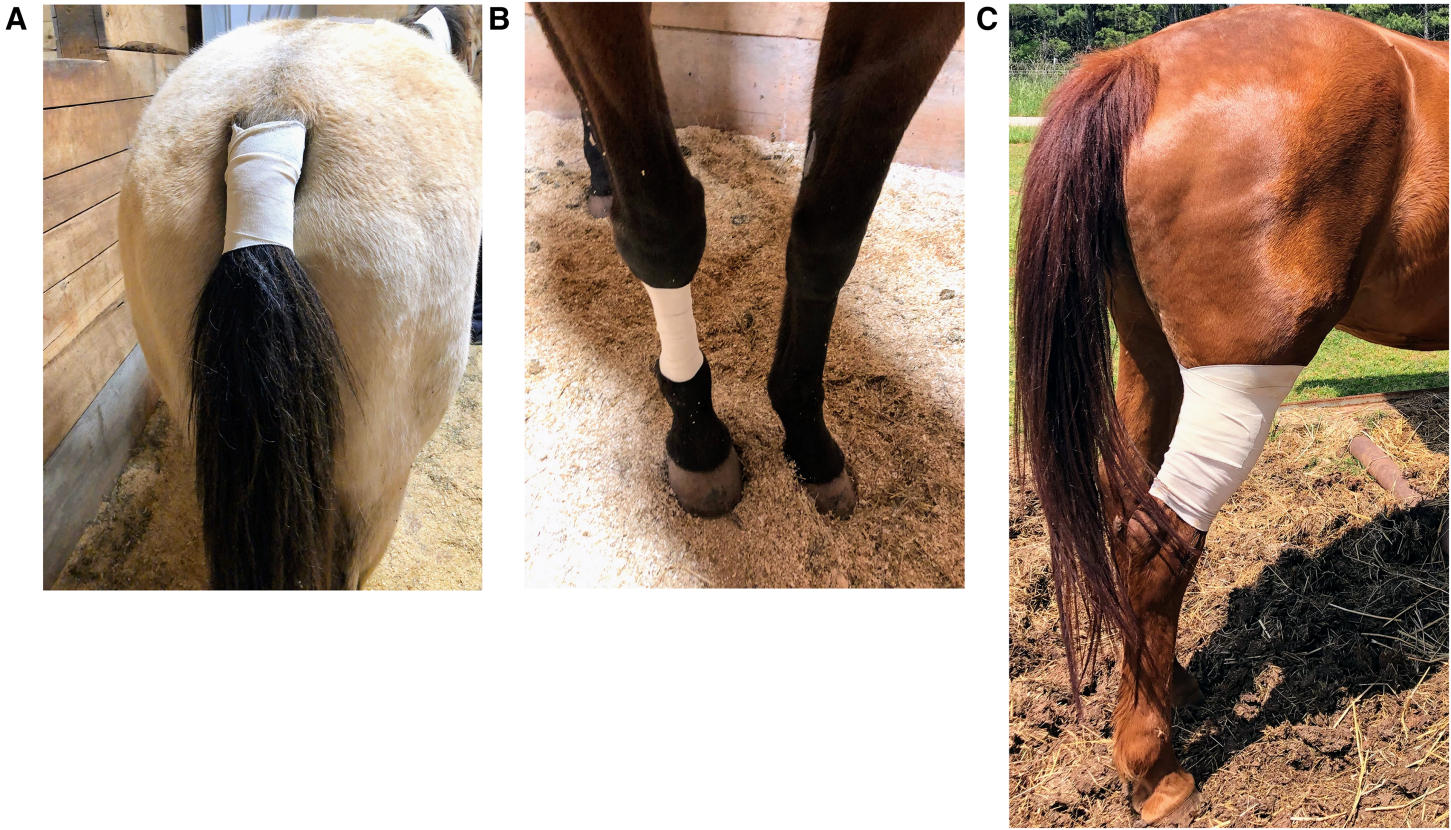
Placement of two transdermal matrix-type patch systems each containing 20 mg total buprenorphine (20 μg h^−1^; Amneal Pharmaceuticals LLC., Piscataway, NJ, USA) with dimensions 74 mm × 74 mm and further secured with a 7.62 cm porous elastic adhesive tape covering (Elastikon; Johnson & Johnson, New Brunswick, NJ, USA) on the ventral aspect of the tail base. BUP0 horses did not receive a patch, instead only the elastic adhesive tape was wrapped around the tail base. Hence, the total content was 40 mg, and the dose received was 0.07–0.09 μg kg^−1^ h^−1^ based on their body weights on the day of treatment. The three selected patch locations were as follows: (**A**) Tail_TDP_, patch application to the ventral aspect of the tail base; (**B**) Metacarpus_TDP_, patch application to the dorsal surface of the metacarpus; and (**C**) Gaskin_TDP_, patch application to the gaskin region located between stifle and hock joints.

All horses in our study were administered to each of the following three treatment groups, and the randomization by application of Latin square was predetermined (www.randomizer.org). The washout period between treatments was a minimum of 10 days. The hair was clipped over the location of interest using a #50 clipper blade as required to allow enough area for two patches placed alongside each other in a vertical arrangement without overlap and adequate patch-to-skin contact was ensured. The clipped area was then wiped clean with a dry 10.16 cm × 10.16 cm gauze pad to remove dirt and skin debris. Two transdermal patches, each containing 20 mg total buprenorphine (20 μg h^−1^; Amneal Pharmaceuticals LLC., Piscataway, NJ, USA) with dimensions 74 mm × 74 mm, were applied to the assigned location using their adhesive surface and were further secured with a 7.62 cm porous elastic adhesive tape covering (Elastikon; Johnson & Johnson, New Brunswick, NJ, USA) as shown in Figure 1. Hence, the dose received was 0.07–0.09 μg kg^−1^ h^−1^ based on their body weights. The firm adherence of the patch at the location was confirmed by visual inspection at each data and blood collection timepoint. The three selected locations were as follows:

1. **Tail_TDP_:** patch application to the ventral aspect of the tail base (Figure 1A)
2. **Metacarpus_TDP_:** patch application to the dorsal surface of the metacarpus (Figure 1B)
3. **Gaskin_TDP_:** patch application to the gaskin region located between stifle and hock joints (Figure 1C)

### 2.4 Study timeline and data collection

**Figure 2:**
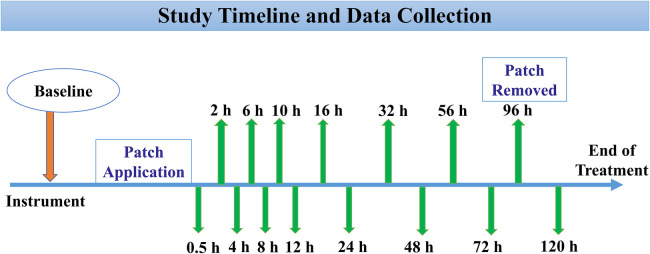
Following instrumentation, baseline data (0 h) was acquired consisting of physical examination and collection of jugular blood samples. Depending on the patch location (ventral aspect of the tail base, metacarpal, and gaskin region), the treatment was initiated by placing two transdermal matrix-type patch systems each containing 20 mg total buprenorphine (20 μg h^−1^; Amneal Pharmaceuticals LLC., Piscataway, NJ, USA). Hence, the total content was 40 mg, and the dose received was 0.07–0.09 μg kg^−1^ h^−1^ based on their body weights on the day of treatment. Following this, 6 ml whole blood samples were obtained for determination of buprenorphine plasma concentration at 0.5, 2, 4, 6, 8, 10, 12, 16, 24, 32, 48, 56, 72, 96, and 120 h after the patches were applied. The transdermal patches were removed at the 96 h timepoint. The last data collection for physiologic variables and blood sampling was performed at 120 h, which marked the end of data collection for that treatment.

The entire timeline of the study during administration of a treatment is depicted in Figure 2. Following instrumentation for the IV catheter, baseline data (0 h) consisting of HR, RR, and Temp_rectal_ was acquired along with a collection of 6 ml whole blood from the jugular catheter. On the treatment day, each horse underwent patch application in the location designated by the randomization. Following application, additional whole blood samples were obtained for determination of buprenorphine plasma concentration at 0.5, 2, 4, 6, 8, 10, 12, 16, 24, 32, 48, 56, 72, 96, and 120 h after the patches were applied. A 10 ml waste sample was procured from the jugular catheter before drawing the 6 ml sample of venous blood for buprenorphine plasma concentrations. The sampling jugular catheter was removed after 72 h, and the following blood samples were obtained by direct jugular venipuncture. The transdermal patches were also removed at the 96 h timepoint. They were collected in sterile bags and stored at −80°C until later analysis of residual buprenorphine content. The last data collection for physiologic variables and blood sampling was performed at 120 h, which marked the end of data collection for that treatment. Blood samples were collected in lithium heparin tubes (Green BD Hemogard; Becton-Dickinson, Franklin Lakes, NJ, USA) and immediately underwent centrifugation at 1,300×*g* for 10 min. The resultant supernatant plasma was aspirated via a 1 ml disposable pipette (Thermo Fisher Scientific, Waltham, MA, USA) and transferred to cryogenic vials (Labcon 1.5 ml SuperSpin; Thermo Fisher Scientific), which were then stored at −80°C until analysis (within 2 months of sample collection).

### 2.5 Determination of buprenorphine concentrations

Plasma calibrators were prepared by dilution of the buprenorphine working standard solution (Cerilliant, Round Rock, TX, USA) with drug-free equine plasma to concentrations ranging from 0.01 to 70 ng ml^−1^. Calibration curves, negative control samples, and quality control samples were freshly prepared for each assay. Quality control samples (drug-free equine plasma fortified with buprenorphine) were prepared at 0.15, 4.0, and 40 ng ml^−1^ and were included with each sample set.

For drug extraction, 0.5 ml of plasma samples were diluted with 2.0 ml 0.1M pH 6 phosphate buffer and 0.1 ml water containing d4-buprenorphine as the internal standard (40 ng ml^−1^; Cerilliant, Round Rock, TX). All samples were vortexed gently to mix and subjected to solid phase extraction using C18UC columns 200 mg 3 ml^−1^ (UCT, Bristol, PA, USA). Prior to the addition of the samples, the columns were conditioned with 2.5 ml of methanol and 3 ml of water. Samples were loaded onto the column, and a minimum of 2 min was allowed for samples to pass through the column. The columns were rinsed with 2 ml 50% methanol in water, prior to eluting with 2.5 ml methanol. Samples were then dried under nitrogen, dissolved in 120 µl of 10% acetonitrile (ACN) in water with 0.2% formic acid, and 40 µl injected into the liquid chromatography-tandem mass spectrometry (LC-MS/MS) system.

Buprenorphine concentrations were measured in plasma by LC-MS/MS using positive heated electrospray ionization HESI (+). A TSQ Altis triple quadrupole mass spectrometer coupled with a Vanquish liquid chromatography system (Thermo Scientific, San Jose, CA, USA) was used for quantitative analysis. Product masses and collision energies were optimized by infusing the analytes into the mass spectrometer. Chromatography employed an ACE 3 C18 10 cm × 2.1 mm 3 µm column (Mac-Mod Analytical, Chadds Ford, PA, USA) and a linear gradient of ACN in water with a constant 0.2% formic acid at a flow rate of 0.4 ml min^−1^. The initial ACN concentration was held at 10% for 0.3 min, ramped to 95% over 4.6 min, and held at that concentration for 0.3 min, before re-equilibrating for 2.8 min at initial conditions.

Detection and quantification were conducted using selective reaction monitoring (SRM) of the initial precursor ion for buprenorphine [mass to charge ratio (*m/z*) 468.3] and the internal standard d4-buprenorphine [(*m/z*) 472.3]. The response for the product ions for buprenorphine (*m/z* 101.0, 186.9, 243.0, 396.2, 414.2) and the internal standard (*m/z* 100.9, 186.9) were plotted, and peaks at the proper retention time-integrated, using Quan Browser software (Thermo Fisher Scientific). Quan Browser software was used to generate calibration curves and quantify the analyte in all samples by linear regression analysis. A weighting factor of 1/X was used for all calibration curves.

The patches were cut into 1 cm^2^ portions and divided into two 50 ml plastic tubes. Tubes were extracted three times with 30 ml methanol by rotating for 30 min and sonicating for 5 min. The extracts were combined, brought to a final volume of 200 ml with methanol, and 200 µl was subsequently diluted to 2 ml with methanol. An aliquot (100 µl) was subjected to solid phase extraction as described for the plasma samples, and 20 µl was injected into the LC-MS system using the analytical conditions described previously.

The response for buprenorphine was linear and gave correlation coefficients of 0.99, or better accuracy was reported as percent nominal concentration and precision were reported as percent relative standard deviation. Accuracy was 98% for 0.15 ng ml^−1^, 99% for 4 ng ml^−1%^, and 104% for 40 ng ml^−1^. Precision was 5% for 0.15 ng ml^−1^, 2% for 4 ng ml^−1^, and 2% for 40 ng ml^−1^. The technique was optimized to provide a limit of quantitation of 0.01 ng ml^−1^ and a limit of detection of approximately 0.005 ng ml^−1^ for buprenorphine.

### 2.6 Data analysis

Numerical data such as HR, RR, and Temp_rectal_ were assessed for normality using the Shapiro–Wilk test and by observing histograms and normal Q-Q residual plots. Mixed-effects two-factor analysis of variance was used to interpret the effects of time and treatment (fixed nominal effects) and the association of horse-time and horse-treatment was added as random effects. To adjust for the lack of sphericity, the Greenhouse–Geissner correction was applied. For making multiple comparisons with baseline measurements, the *post hoc* Tukey honest significant difference test and Dunnett's test were conducted. For all analyses (SAS 9.4; SAS Institute Inc., Cary, NC, USA), *p* < 0.05 was considered statistically significant.

## 3 Results

All horses successfully completed the study, and patch application was well tolerated in all three locations. Application sites were observed at each timepoint to ensure the patches were intact and in good contact with the skin. In one horse, the patches did not adhere well at the gaskin region, resulting in missing data from the 48 h timepoint until the last timepoint. Upon patch removal, there was no evidence of skin inflammation, papules, skin irritation, or redness. All horses remained clinically healthy throughout the study, and no clinically apparent adverse effects were noted with the buprenorphine dose during the entire study period. Based on the subjective data during physical examination, no horse showed signs of colic or central nervous system excitation with the dose used. Overall, the horses cooperated well and stood quietly using a halter with lead rope restraint while the physical examination was being conducted.

### 3.1 Physical examination

The physical examination variables followed a normal distribution, and hence the values are represented as mean ± standard deviation. The HR at the baseline timepoint for Tail_TDP_, Metacarpus_TDP_, and Gaskin_TDP_ was 38 ± 4, 39 ± 3 and 41 ± 3 beats/min, respectively. The RR at the baseline timepoint for Tail_TDP_, Metacarpus_TDP_, and Gaskin_TDP_ was 22 ± 3, 19 ± 4, and 21 ± 3 breaths/min, respectively. The Temp_rectal_ at baseline timepoint for Tail_TDP_, Metacarpus_TDP_, and Gaskin_TDP_ was 98.9 ± 0.84, 99.9 ± 0.93, and 99.5 ± 0.98°F, respectively. Between the three treatment groups, there was no change in HR, RR, and Temp_rectal_ across timepoints as compared to baseline and when compared to each other in a single horse as well as between horses (*p* > 0.3). There was no effect of treatment (*p* > 0.2) or time (*p* > 0.1) and no significant interaction between treatment and time on HR, RR, and Temp_rectal_.

### 3.2 Plasma buprenorphine concentrations

**Figure 3:**
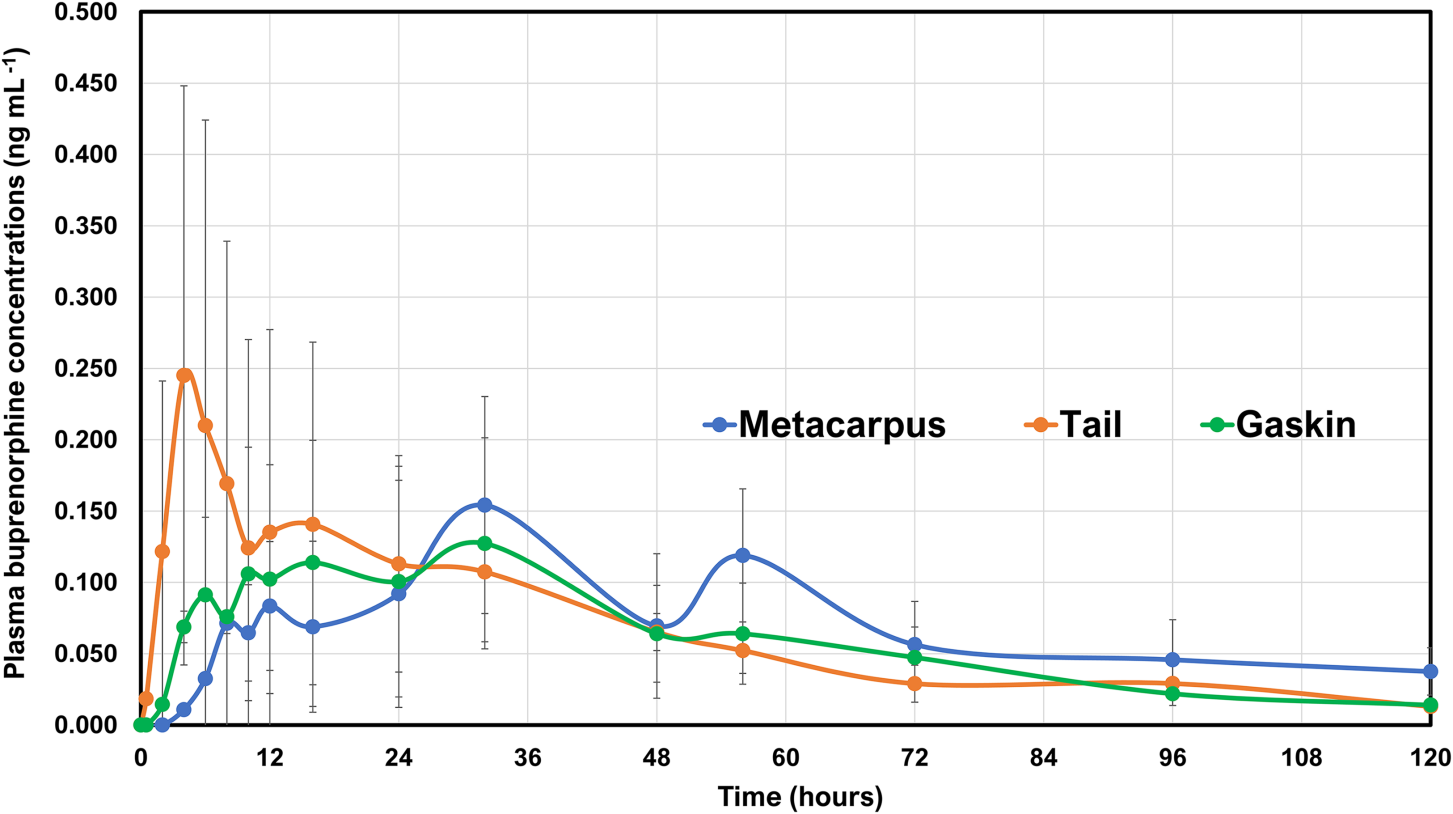
Mean ± standard deviation of plasma concentrations of buprenorphine overtime in six horses from baseline (0 h), which coincides with before patch application to 0.5, 2, 4, 6, 8, 10, 12, 16, 24, 32, 48, 56, 72, 96, and 120 h after the patches were applied. Two transdermal matrix-type patch systems each containing 20 mg total buprenorphine (20 μg h^−1^; Amneal Pharmaceuticals LLC., Piscataway, NJ, USA) were placed in three different locations. Tail, patch application to the ventral aspect of the tail base (orange lines with orange circles); Metacarpus, patch application to the dorsal surface of the metacarpus (blue line with blue circles); and Gaskin, patch application to the gaskin region located between stifle and hock joints (green line with green circles). The total content was 40 mg, and the dose received was 0.07–0.09 μg kg^−1^ h^−1^ based on their body weights on the day of treatment. The transdermal patches were removed at the 96 h timepoint.

In the Tail_TDP_ group, the mean plasma buprenorphine concentrations were >0.1 ng ml^−1^ from 2 h to 32 h. The highest group mean was 0.25 ng ml^−1^ noted at 4 h. In the Metacarpus_TDP_ group, the mean plasma buprenorphine concentrations were >0.1 ng ml^−1^ from 32 to 56 h. The highest group mean was 0.15 ng ml^−1^ noted at 32 h. In the Gaskin_TDP_ group, the mean plasma buprenorphine concentrations were >0.1 ng ml^−1^ from 10 to 32 h. The highest group mean was 0.13 ng ml^−1^ noted at 32 h. Out of the total six horses, one horse in the Tail_TDP_ group, five horses in the Metacarpus_TDP_ group, and four horses in the Gaskin_TDP_ group had detectable plasma buprenorphine concentrations at the 120 h timepoint. Norbuprenorphine was not detected in any horse at concentrations above the limits of detection at any time point. When comparing all three locations, the buprenorphine uptake was observed to be more consistent with respect to measurable plasma concentrations >0.1 ng ml^−1^ when applied to the ventral aspect of the tail base (Figure 3).

When the patches were removed and submitted for analysis, the amount of buprenorphine extracted from patches was 23 ± 1.5 mg (54.5 ± 4.3% left) in the Tail_TDP_ group, 21.1 ± 2.3 mg (55.6 ± 6.5% left) in the Metacarpus_TDP_ group, and 23.4 ± 2.5 mg (58 ± 5.3% left) in the Gaskin_TDP_ group.

## 4 Discussion

For the present study, the aim was to determine buprenorphine plasma concentrations in healthy horses from transdermal patches applied at three different locations i.e., the ventral aspect of the tail base, the metacarpal region, and the gaskin area. Skin preparation and the process of patch application were followed as per the standard published in other equine studies to maintain uniformity in the technique (26, 27, 33–36). The plasma buprenorphine concentrations were consistently >0.1 ng ml^−1^ as quickly as 2 h and lasted up to 32 h for the Tail_TDP_ group. Although the other two locations yielded measurable plasma concentrations, they were >0.1 ng ml^−1^ at fewer timepoints. The drug was detected faster in the plasma and a higher peak was observed in the Tail_TDP_ group. In our horses, the desired level for plasma buprenorphine concentration was set at a minimum of 0.1 ng ml^−1^ and was based on a recent study (26) that showed the placement of two transdermal buprenorphine patches (each containing 20 mg total buprenorphine) on ventral tail base resulted in a consistent increase in thermal thresholds that coincided with ≥0.1 ng ml^−1^ in healthy horses. To the author's knowledge, the present study is the first to report buprenorphine plasma concentrations in horses following patch application at different locations.

Transdermal opioid delivery systems have gained immense popularity across different species, which has contributed to significant advances in effective pain management via the maintenance of steady blood drug concentrations over longer periods. The established transdermal opioid delivery systems are drug-in-adhesive, reservoir, and matrix-type. In the present study, buprenorphine was administered via a matrix patch that includes an adhesive polymer matrix containing the drug homogeneously embedded in the center. On the top of this matrix is the backing layer made up of elastomers that protect the patch from the outer environment and it is impermeable to the drug. On the bottom of this matrix is the lining layer that protects the patch during storage and is peeled off before use (3, 4, 37, 38). The matrix-type patch is relatively thinner, lighter, and flexible, which benefits skin conformability and adherence. The thickness of the adhesive polymer matrix layer indicates that some of the drug will diffuse through the layer before reaching the skin. This design enables the drug to get across the dermis to the cutaneous blood vessels for absorption into circulation where it becomes available systemically. If the active form of the drug remains largely in the periphery, there is limited penetration into the systemic circulation, which reduces the incidence of adverse effects (3, 4). Adherence of the patch with the skin is crucial for the efficacy of this transdermal delivery system. Skin and body movement, rubbing the patch subject the patch to sheer stress impacting adhesion. Moreover, environmental factors such as sweating, moisture, and ambient temperature have a direct effect on patch-to-skin contact. It is possible that these mechanisms may have played a role in the present study and contributed to the inconsistent or lower plasma buprenorphine concentrations for patches placed on the gaskin and metacarpal regions.

Skrzypczak et al. (27) applied matrix-type fentanyl patches to the inguinal abdominal region (lateral to udder or prepuce), dorsal metacarpus, and ventral aspect of the tail base in healthy horses. They observed that the maximum fentanyl concentration and the time taken to reach this drug concentration were similar between locations. The patches were well tolerated at these sites and no treatment was affected by the loss of patch via dislodgement. The other locations that have been studied to evaluate reservoir-type fentanyl patches in horses are the proximal lateral antebrachium (33), medial or lateral antebrachium and gaskin region (34), and mid-dorsal thorax (35). There is a significant location-dependent difference in transdermal fentanyl penetration in horses (27, 32), sheep (28), and rabbits (29), with less drug available for the systemic activity for patches applied to the dorsal carpal region in horses, whereas the groin and thorax skin have a similar pattern (32). Several factors can account for species-specific differences and inter-patient variability with respect to drug uptake from the patch and absorption via the skin such as (i) thickness of stratum corneum and epidermis, (ii) density of hair follicles and sweat glands, (iii) regional cutaneous blood flow, (iv) drug molecular kinetics, (v) genetics, (vi) underlying skin disease or injury, (vii) formulation of the drug–polymer matrix, (viii) skin temperature, and (ix) skin preparation (razor shaving, alcohol). Fick's law of diffusion controls the rate of drug input from the transdermal system into the systemic circulation through skin penetration barriers, where the drug delivery is directly proportional to the drug concentration in the matrix and coefficient of drug diffusion. It is vital to note that the drug penetration into the skin is not constant and is dependent on the duration of patch application and overtime variations in cutaneous properties, available drugs in the matrix, and depletion of enhancers required for drug delivery (3, 32). In the present study, cleaning the application site could have disrupted the stratum corneum, and not all drug from the patch was delivered while in contact with the skin. Erratic drug uptake between locations could have been a consequence of altered diffusion capacity of the skin lipids, differences in skin thickness, and variations in skin pH due to sweat, moisture, and altering body temperature. In one horse belonging to the Gaskin_TDP_ group, the patches were seen to not firmly adhere due to sweat, moisture, and leg movement and the bandage tended to slip down in that area. This finding is clinically relevant and should be taken into consideration when using this patch location.

Special features that ease the crossing of buprenorphine through the skin are lower molecular weight, compact molecular structure, high lipophilicity, an adequate degree of ionization, sufficient water solubility, high efficacy to restitute for limited absorption, reduced melting temperature, relatively shorter half-life, low daily dosage regime, dosing enabling absorption from a relatively small area, and matrix patches in which a total amount of a drug is localized homogenously in an adhesion layer (3, 5–7). This technology ensures the release of the opioid is regulated due to the gradient concentration between the patch and the skin. Patch delivery systems are designed particularly to contain more amount of drugs than the patch actually can deliver. In the event the patch is not removed from the location, increased dose administration and prolonged pharmacological effects can occur. We removed the patch from the horses at the 96 h timepoint, and the residual drug was determined. The buprenorphine residue on the patch was 21–23 mg (54%–58%) of the total amount (40 mg). The residual amount can be influenced by the type of patch, drug load and concentration, the thickness of the adhesive layer, and the composition and thickness of the backing layer. Although this can be a safety concern with the potential for abuse, the excess amount of the drug remaining in the patch after use is necessary to ensure a saturated concentration of the drug is maintained and drug delivery occurs at a clinically effective rate. The development of metered-dose pumps or active diffusion systems may prove beneficial to increase drug efficiency and improve safety or abuse liability profiles. Poor patch-to-skin contact and variable skin hydration can occur in response to ambient humidity and temperature and affect the integrity and barrier properties of the skin resulting in variations in the amount of drug absorbed (37, 38). The 54%–58% buprenorphine left over in the patch explains why we saw lower plasma concentrations and, hence, did not observe any significant behavioral effects and differences in the physical examination. However, it also signifies that despite partial drug uptake, the plasma concentrations obtained were >0.1 ng ml^−1^ for multiple timepoints in the Tail_TDP_ group and relatively fewer timepoints for the Metacarpus_TDP_ and Gaskin_TDP_ groups.

The primary metabolite of buprenorphine is norbuprenorphine, which was undetectable following transdermal administration in the present study. This analysis was in accordance with previous studies where norbuprenorphine was unmeasurable following either intravenous or sublingual route (39–41). Considering norbuprenorphine has only 25% of the intrinsic analgesic activity of buprenorphine and a low permeability into the brain, it may have minimal clinical significance (42). There is no available literature highlighting the antinociceptive effect of norbuprenorphine in horses and hence it is uncertain whether this metabolite contributes to antinociception. It is possible that the high stability of molecular ions of norbuprenorphine may present a challenge to be detected by tandem mass spectrometry. The assay may not have the sensitivity for measuring this metabolite and this lack of optimization could affect this finding.

Previous exploratory studies with buprenorphine in horses utilized average doses of 5–10 µg/kg via intravenous (18, 20, 21, 40, 42–48), intramuscular (23, 24, 39, 49), and sublingual (40, 42, 50) routes. A common observation in most of these studies irrespective of the route used was its potential for inducing excitement, increasing spontaneous locomotory activity, decreasing gut sounds, and elevating HR in healthy pain-free horses. Despite opting for the subcutaneous route for buprenorphine administration in a few equine studies, the gastrointestinal side effects, compulsive behavior, and restlessness persisted (22, 51). The dose in the present study was selected carefully based on the behavioral and physiologic responses reported in these studies. We anticipated that 0.07–0.09 μg kg^−1^ h^−1^ (40 μg h^−1^) would be a safe, well-tolerated dosage regime for our horses, which would prevent systemic complications and excitement as confirmed in the previous equine studies (25, 26). Moreover, currently, the highest concentration of transdermal system available for buprenorphine in the USA is 20 μg h^−1^, and since the selected locations were the ventral aspect of the tail base, metacarpal and gaskin regions, placement of only two patches next to each was possible without overlap to administer 40 μg h^−1^. Future studies are imperative to evaluate whether a higher transdermal patch dose can lead to plasma concentrations lasting for a longer duration coinciding with therapeutic drug concentrations yielding adequate analgesia but still devoid of any systemic complications. In addition, even though mild, diffuse erythema with a small number of papules has been reported with buprenorphine transdermal system in pigs (13), no adverse effects were noted locally near or at the area of patch location in our study horses.

This study presented a few limitations. An intravenous treatment was not included in the study design, and therefore, the bioavailability of the matrix buprenorphine patch was not calculated. Only a small sample size consisting of healthy, pain-free adult horses was utilized. The physiologic and behavioral effects of opioid administration can differ significantly in painful vs. non-painful animals; hence, future studies in clinical patients exhibiting signs of pain are warranted. A genetic involvement for transdermal drug uptake has been defined in humans; however, its impact cannot be ruled out in our study of horses. Aging induces structural and functional variations in the skin layers and changes in hydration and lipidic structure may affect the barrier function of the stratum corneum specially for hydrophilic compounds. Hence, potential alterations affecting the transdermal opioid diffusion in younger vs. older horses need further investigation. Noxious thermal stimuli to evaluate the analgesic effect of transdermal buprenorphine patches at various locations for superficial acute short-lasting pain were not included. The minimum therapeutic levels for buprenorphine via this route remain unknown. Behavioral analysis and gastrointestinal function were not assessed using standards published in the literature (e.g., video footage, pedometer data, gastrointestinal motility scores, fecal and urine output, visual analog scoring, ataxia grading, and sedation scores). Since the undesirable effects can be of lesser magnitude in painful horses, future clinical studies are required that objectively quantify these effects and determine their association with transdermal buprenorphine patch administration in painful vs. non-painful horses.

## 5 Conclusion

Following extensive literature review, this appears to be one of the earlier reports of transdermal buprenorphine patch administration in horses. In the present study, 40 μg h^−1^ buprenorphine transdermal patches applied at the ventral aspect of the tail base, metacarpal, and gaskin region were well tolerated by all horses as assessed by a physical examination. In the Tail_TDP_ group, the mean plasma buprenorphine concentrations were >0.1 ng ml^−1^ from 2 to 32 h. The highest group mean was 0.25 ng ml^−1^ noted at 4 h. In the Metacarpus_TDP_ group, the mean plasma buprenorphine concentrations were >0.1 ng ml^−1^ from 32 to 56 h. The highest group mean was 0.15 ng ml^−1^ noted at 32 h. In the Gaskin_TDP_ group, the mean plasma buprenorphine concentrations were >0.1 ng ml^−1^ from 10 to 32 h. The highest group mean was 0.13 ng ml^−1^ noted at 32 h. Norbuprenorphine was not detected in any horse at concentrations above the limits of detection at any time point. When comparing all three locations, the buprenorphine uptake was observed to be more consistent with respect to measurable plasma concentrations >0.1 ng ml^−1^ when applied to the ventral aspect of the tail base. The other two locations presented more erratic and inconsistent buprenorphine uptake and plasma concentrations. Further research must be directed at investigating the effect of higher dosages of the transdermal buprenorphine patch on the duration of analgesia, measurable plasma concentrations, and behavioral and systemic effects. It is imperative that clinicians can compare analgesic and systemic effects in painful and non-painful horses.

## Data Availability

The original contributions presented in the study are included in the article/Supplementary Material; further inquiries can be directed to the corresponding author.
